# First Documented Atrial Fibrillation Triggered by Dialysis-Induced Autonomic Instability in End-Stage Kidney Disease

**DOI:** 10.7759/cureus.106559

**Published:** 2026-04-06

**Authors:** Yasir Kammawal, Matiullah Azmoon

**Affiliations:** 1 Internal Medicine, Isle of Wight NHS Trust, Isle of Wight, GBR; 2 Cardiology, Bahawal Victoria Hospital, Bahawalpur, PAK

**Keywords:** amiodarone, arrhythmia, atrial fibrillation, autonomic instability, end-stage kidney disease, hemodialysis

## Abstract

Atrial fibrillation (AF) is the most common sustained arrhythmia in the general population. However, it is seldom the initial rhythm disturbance in dialysis-dependent patients, who more often present with bradyarrhythmias or ventricular tachyarrhythmias. We report a 59-year-old man with end-stage kidney disease on thrice-weekly hemodialysis who developed his first documented AF with rapid ventricular response immediately after dialysis. Coexisting hypotension, asthma, and severely reduced left ventricular ejection fraction limited the use of β-blockers, calcium channel blockers, and digoxin. Given these limitations, intravenous amiodarone was selected as the most appropriate option, providing both rhythm and partial rate control while maintaining hemodynamic stability, and cautious crystalloid resuscitation was administered, resulting in partial stabilization and intermittent reversion to sinus rhythm.

This case illustrates dialysis as a direct arrhythmogenic stressor through autonomic imbalance and electrolyte shifts, with underlying ventricular dysfunction serving as a substrate. It emphasizes the challenges of managing AF when standard therapies are contraindicated and highlights the importance of individualized treatment in dialysis patients with complex comorbidities.

## Introduction

Patients with end-stage kidney disease (ESKD) on chronic hemodialysis are prone to arrhythmias, a major cause of cardiovascular morbidity and mortality in this population. Although atrial fibrillation (AF) is the most common sustained arrhythmia in the general population, it has been reported in approximately 13-27% of patients on chronic hemodialysis. However, available studies suggest that peri-dialytic arrhythmic events are more commonly bradyarrhythmic, with ventricular arrhythmias also reported, and AF is less frequently emphasized as the initial rhythm disturbance in this setting [1-3].

Dialysis induces rapid shifts in intravascular volume and electrolytes, accompanied by significant autonomic stress. A mismatch between sympathetic activation and impaired parasympathetic recovery has been identified as a mechanism that promotes atrial ectopy and arrhythmogenesis [2]. Additional factors, such as fluctuations in potassium, calcium, and magnesium, further destabilize atrial conduction.

Most studies of AF in dialysis emphasize its chronic prevalence and the difficulties of anticoagulation [3]. By contrast, the role of acute autonomic triggers in precipitating AF immediately after dialysis has received limited attention.

We present a case of the first documented AF occurring immediately after dialysis in a patient with asthma and severe heart failure with reduced ejection fraction (HFrEF), where management was complicated by limited therapeutic options.

## Case presentation

**Figure 1:**
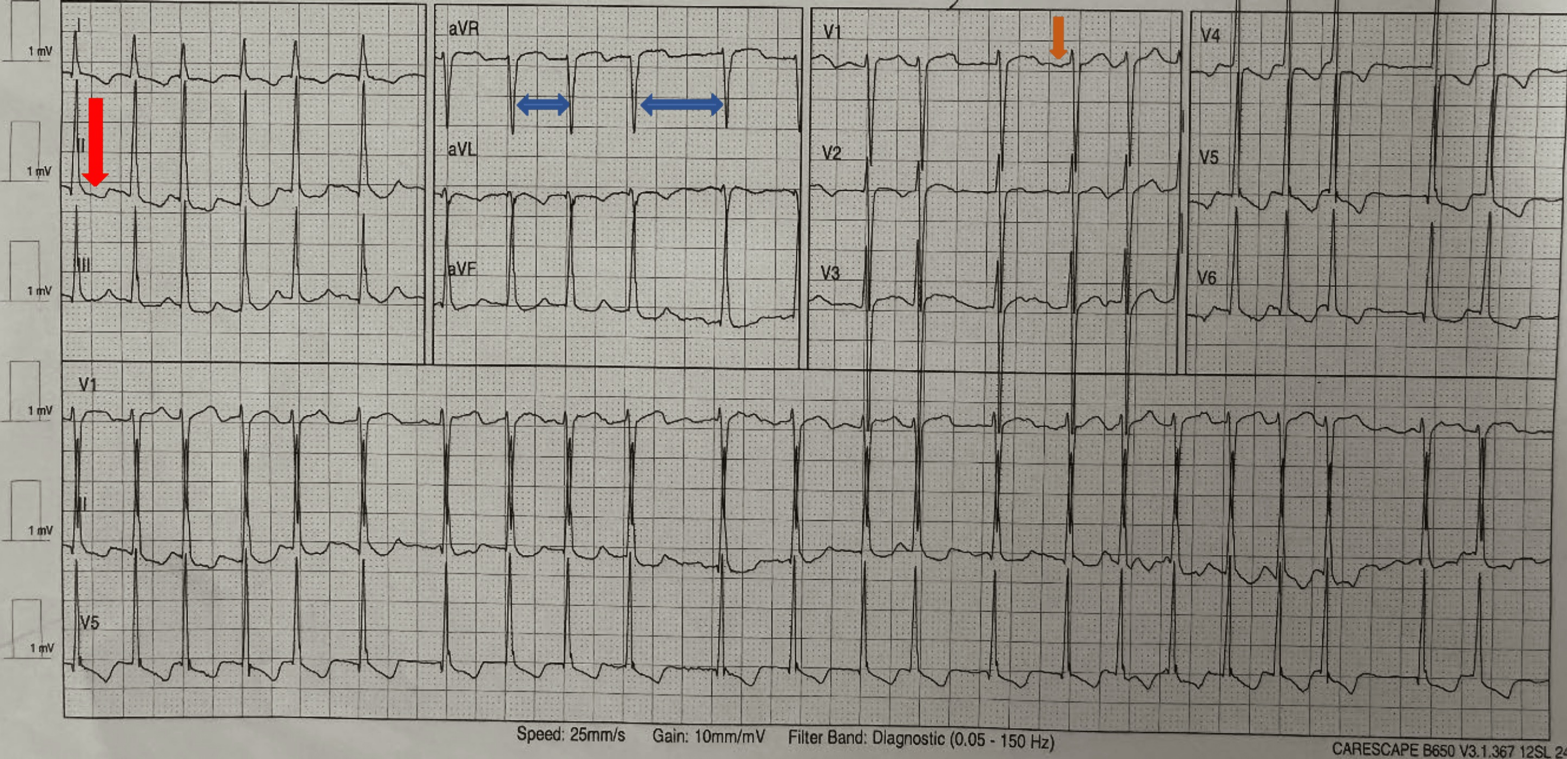
Electrocardiogram showing atrial fibrillation with rapid ventricular response (~140 bpm), irregular R-R intervals, fibrillatory waves, and rate-related ST depression in the inferolateral leads. The blue arrows indicate the irregular R-R intervals characteristic of atrial fibrillation. The orange arrow highlights fibrillatory waves, most evident in lead V1. The red arrow marks rate-related ST depression in the inferolateral leads.

A 59-year-old man with ESKD on thrice-weekly hemodialysis, hypertension, asthma, and HFrEF (27%) presented to the emergency department after a fall at home. He had just completed a dialysis session and reported dizziness and lightheadedness on standing but denied chest pain, palpitations, respiratory, gastrointestinal, or urinary symptoms, with no history of syncope or trauma. On further questioning, he described experiencing similar post-dialysis dizziness in the past, which he had assumed were expected effects of dialysis and had not sought medical care. On this occasion, the symptoms were more severe, resulting in a fall. An electrocardiogram (ECG) performed at triage revealed AF (Figure 1).

On arrival, he was alert and oriented but hypotensive, with blood pressure ranging from 80 to 95/60 to 70 mmHg, heart rate varying between 85 and 135 beats per minute (irregularly irregular), and oxygen saturation of 97% on room air. Cardiovascular examination revealed an irregular rhythm, fine bibasal crepitations, and mild pedal edema. Respiratory examination was clear, with no audible wheeze.

Laboratory results on admission are summarized in Table 1. Sepsis screening, including blood cultures, was initiated at admission, with results pending at that time. Troponin results were pending at admission.

**Table 1:** Laboratory findings at admission. Admission laboratory results demonstrating anemia, inflammation, electrolyte shifts, and reduced kidney function consistent with ESKD. ESKD: end-stage kidney disease; eGFR: estimated glomerular filtration rate.

Test	Result	Reference range	Comment
Hemoglobin	113 g/L	130-170 g/L (male)	Mild anemia
White cell count	10.1 × 10⁹/L	4.0-11.0 × 10⁹/L	Slightly elevated
C-reactive protein	54 mg/L	<5 mg/L	Elevated
Sodium	132 mmol/L	135-145 mmol/L	Low-normal
Potassium	5.0 mmol/L (pre-dialysis 5.7)	3.5-5.0 mmol/L	Improved post-dialysis
Urea	24 mmol/L (pre-dialysis 32)	2.5-7.0 mmol/L	Consistent with ESKD
Creatinine	463 μmol/L	60-110 μmol/L	Markedly elevated
eGFR	5 mL/min/1.73 m²	>90 mL/min/1.73 m²	Severe impairment
Calcium	2.05 mmol/L	2.10-2.60 mmol/L	Low-normal
Magnesium	0.70 mmol/L	0.70-1.00 mmol/L	Lower end of normal
Troponin	Pending at admission	<14 ng/L (high-sensitivity)	—

In view of hypotension, asthma, and severe left ventricular (LV) dysfunction, β-blockers and calcium channel blockers were limited by hypotension, while asthma prompted additional caution with β-blocker therapy. Digoxin was avoided due to the risk of accumulation and toxicity in ESKD [4,5]. The patient was commenced on intravenous amiodarone (300 mg over 60 minutes, followed by 900 mg over 24 hours), which achieved partial rate control. A cautious 150 mL crystalloid bolus was administered to improve perfusion while minimizing the risk of post-dialysis fluid overload. Clinical evaluation and initial investigations revealed no immediate evidence of an infectious source. Thyroid function tests were also sent.

Over the next 24 hours, the patient stabilized, with intermittent reversion to sinus rhythm and improved hemodynamics. He remained under joint cardiology and nephrology review. Given that this was his first documented episode of AF, the decision regarding long-term anticoagulation was deferred pending multidisciplinary evaluation of stroke and bleeding risk. Long-term follow-up data were not available at the time of writing.

## Discussion

This case highlights the first documented episode of AF occurring immediately following hemodialysis. In dialysis patients, arrhythmias are more often ventricular or bradyarrhythmic, and although AF has been reported in approximately 13-27% of patients on chronic hemodialysis, it is less frequently described as the initial arrhythmic event occurring immediately post-dialysis [1,3]. Hemodialysis is associated with rapid intravascular volume shifts that stimulate baroreceptors and provoke sympathetic activation, while parasympathetic recovery is blunted, creating an autonomic imbalance [2]. This autonomic stress increases atrial ectopy and susceptibility to AF, as autonomic imbalance has been shown to act as both a trigger and substrate for AF in physiological studies, with additional evidence highlighting the role of renal sympathetic activation and cardiorenal autonomic cross-talk in AF initiation [2,6,7].

This concept is further supported by device-based monitoring studies demonstrating that the dialysis procedure itself may act as a trigger for AF through hemodynamic and autonomic changes, even in patients with pre-existing AF [8,9]. Electrolyte fluctuations, particularly changes in potassium and calcium, further destabilize atrial conduction [2]. In this case, the post-dialysis reduction in serum potassium and the relatively low-normal calcium level may have contributed to increased atrial excitability and electrical instability, thereby facilitating AF in the setting of autonomic stress. Severe LV dysfunction likely provided a background predisposition through atrial stretch and fibrosis; however, the temporal association suggests dialysis-induced autonomic instability as a plausible precipitating trigger. The abnormalities in laboratory results (Table 1) and the rhythm seen on ECG (Figure 1) correlate with these mechanisms. Notably, the patient reported recurrent post-dialysis dizziness in the past, raising the possibility of prior transient or undocumented arrhythmic episodes. However, AF was only confirmed and documented during the current admission when captured on ECG.

Management was especially challenging due to overlapping comorbidities. Hypotension limited the use of β-blockers and calcium channel blockers, while underlying asthma required caution with β-blocker therapy. Digoxin, sometimes considered in hypotensive patients, was avoided because of impaired clearance and toxicity risk in ESKD [5]. Amiodarone was therefore selected as the most appropriate option, providing both rhythm and partial rate control with acceptable hemodynamic stability. Alternative strategies, including cautious use of selective β-blockers, digoxin, and non-pharmacologic approaches such as electrical cardioversion with transesophageal echocardiographic guidance, were considered. However, despite hypotension, the patient remained alert, oriented, and without features of overt end-organ hypoperfusion, suggesting relative hemodynamic stability. Rate control with amiodarone resulted in clinical improvement, and an initial conservative pharmacologic approach was therefore preferred. This approach is consistent with guideline-based management in patients where conventional therapies are limited by hemodynamic instability or comorbidities.

Most literature on AF in dialysis patients emphasizes the challenges of anticoagulation and stroke prevention [3]. Current guideline recommendations remain inconclusive in this population, with limited evidence to support routine anticoagulation and a need for individualized risk-benefit assessment. In addition, pharmacologic cardioversion with agents such as amiodarone may be associated with transient atrial stunning and a potential increase in early thromboembolic risk, which warrants careful consideration of anticoagulation in the immediate period.

By contrast, this case highlights dialysis-induced autonomic instability as a potential and under-recognized trigger for new-onset AF in the immediate post-dialysis period. In our patient, the onset of arrhythmia immediately after dialysis suggests a strong temporal association with this mechanism rather than a coincidental event, although a definitive causal relationship cannot be established from a single observation. This observation is clinically relevant, reminding physicians that dialysis itself can be an arrhythmogenic stressor. Awareness of this possibility may allow earlier recognition and more individualized management of AF in patients otherwise considered at risk primarily for ventricular or bradyarrhythmic events. However, detailed dialysis parameters, including dialysate composition and ultrafiltration volume, were not available, which limits precise characterization of the dialysis-related trigger. Further observational and mechanistic studies are needed to better define the prevalence and underlying pathophysiological mechanisms of dialysis-associated AF.

## Conclusions

We report a case of first documented AF with rapid ventricular response occurring immediately after dialysis in a patient with ESKD. The temporal relationship suggests that dialysis-induced autonomic imbalance, compounded by electrolyte shifts and pre-existing cardiac vulnerability, may have contributed as a precipitating trigger. This case highlights the importance of recognizing dialysis as a potential arrhythmogenic stressor in symptomatic patients, supporting closer ECG monitoring in the post-dialysis period, and underscores the need for individualized, multidisciplinary management when standard therapies are limited by comorbidities. Further observational and mechanistic studies are needed to better define the prevalence and underlying mechanisms of dialysis-associated AF.
